# Crowdsourcing to promote hepatitis C testing and linkage-to-care in China: a randomized controlled trial protocol

**DOI:** 10.1186/s12889-020-09152-z

**Published:** 2020-07-02

**Authors:** William C. W. Wong, Nancy S. Yang, Jingjing Li, Hang Li, Eric Y. F. Wan, Thomas Fitzpatrick, Yuan Xiong, Wai-Kay Seto, Polin Chan, Ruihong Liu, Weiming Tang, Joseph D. Tucker

**Affiliations:** 1 Department of General Practice, HKU-Shenzhen Hospital, Shenzhen, China; 2 Department of Family Medicine and Primary Care, The University of Hong Kong, Hong Kong, China; 3 University of Minnesota Medical School, Minneapolis, MN USA; 4 University of North Carolina Project-China, 1 Global Health Center Office, 2nd Floor of Lao Gan Building, No. 7 Lujing Road, Yuexiu District, Guangzhou City, Guangdong Province, Guangzhou, China; 5 Social Entrepreneurship to Spur Health (SESH), 1 Global Health Center Office, 2nd Floor of Lao Gan Building, No. 7 Lujing Road, Yuexiu District, Guangzhou City, Guangdong Province, Guangzhou, China; 6 Department of Pharmacology and Pharmacy, The University of Hong Kong, Hong Kong, China; 7 University of Washington, Seattle, WA USA; 8 Department of Medicine, The University of Hong Kong, Hong Kong, China; 9 State Key Laboratory for Liver Research, The University of Hong Kong, Hong Kong, China; 10 Department of Medicine, HKU-Shenzhen Hospital, Shenzhen, China; 11 World Health Organization Western Pacific Regional Office, Manila, Philippines; 12 Dermatology Hospital of Southern Medical University, Guangzhou, China; 13 Institute of Global Health and Infectious Diseases, University of North Carolina, Chapel Hill, NC USA

**Keywords:** Hepatitis C virus (HCV), Testing, Primary care, Linkage-to-care, China, Crowdsourcing

## Abstract

**Background:**

Hepatitis C virus (HCV) is a growing public health problem with a large disease burden worldwide. In China many people living with HCV are unaware of their hepatitis status and not connected to care and treatment. Crowdsourcing is a technique that invites the public to create health promotion materials and has been found to increase HIV testing uptake, including in China. This trial aims to evaluate crowdsourcing as a strategy to improve HCV awareness, testing and linkage-to-care in China.

**Methods:**

A randomized controlled, two-armed trial (RCT) is being conducted in Shenzhen with 1006 participants recruited from primary care sectors of The University of Hong Kong-Shenzhen Hospital. Eligible participants are ≥30 years old; a resident in Shenzhen for at least one month after recruitment; no screening for HCV within the past 12 months and not known to have chronic HCV; and, having a WeChat social media account. Allocation is 1:1. Both groups will be administered a baseline and a follow-up survey (4-week post-enrollment). The intervention group will receive crowdsourcing materials to promote HCV testing once a week for two weeks and feedback will be collected thereafter, while the control group will receive no promotional materials. Feedback collected will be judged by a panel and selected to be implemented to improve the intervention continuously.

Those identified positive for HCV antibodies will be referred to gastroenterologists for confirmation and treatment. The primary outcome will be confirmed HCV testing uptake, and secondary outcomes include HCV confirmatory testing and initiation of HCV treatment with follow-ups with specialist providers. Data will be collected on Survey Star^@^ via mobile devices.

**Discussion:**

This will be the first study to evaluate the impact of crowdsourcing to improve viral hepatitis testing and linkage-to-care in the health facilities. This RCT will contribute to the existing literature on interventions to improve viral hepatitis testing in primary care setting, and inform future strategies to improve HCV care training for primary care providers in China.

**Trial registration:**

Chinese Clinical Trial Registry. ChiCTR1900025771. Registered September 7th, 2019, http://www.chictr.org.cn/showprojen.aspx?proj=42788

## Background

Hepatitis C Virus (HCV) is a growing global health problem, with 71 million people estimated living with chronic HCV infection worldwide in 2015 [1]. The global number of deaths due to HCV increased from 333,000 in 1990 to 704,000 in 2013 [2], and it is estimated that HCV-related mortality will triple by 2025 [3]. In China, it is estimated that at least 8.9 million people are living with an identified chronic HCV infection [4]. Notwithstanding, China has the largest burden of liver cancer, accounting to 51% of all global liver cancer death [4]. If untreated, HCV infection can lead to advanced liver diseases, including cirrhosis, liver failure, and hepatocellular carcinoma [5]. In the current era of direct acting antivirals, early testing and treatment can effectively cure HCV and effectively reduce hepatitis-related mortality [6].

Low testing rate and poor linkage to care are key barriers to HCV treatment in China. Since 2018, direct acting antivirals (DAA) regimens such as sofosbuvir/velpatasvir (SOF/VEL) have been included in the national recommended list of essential medications in the China National Formulary (CNF) and have become accessible to patients with a diagnosis. However, only 18% of the HCV-infected population in China had been diagnosed and less than 1.3% actually received treatment [7]. In China, HCV is primarily diagnosed by passive detection, a surveillance system targeting patients seeking care for other illnesses apart from hepatitis, such as enrolled into the hospital for a procedure or surgical intervention, contributing to an underestimation of the actual number of HCV-infected population. Additionally, HCV antibody detection methods and instruments are found to be inconsistent across hospitals. Weakly positive results were considered to be negative, hence, the reported anti-HCV positive rate was slightly lower than the actual level [8]. This may result to an incomplete confirmatory testing since approximately 25–30% of antibody positive will not experience any chronic infection [8].

Promotion of testing and subsequent linkage-to-care service are crucial for effectively curbing the spread of viral hepatitis as well as hepatitis-related morbidity and mortality in China. However, patients are often lost in labyrinth of the Chinese healthcare system and are unable to obtain appropriate screening and treatment. Primary healthcare providers have reported that the lack of motivation and specific training hinder their eagerness to provide HCV testing and treatment to patients, especially in the primary care settings in China [9]. As a result, voluntary HCV testing rates across China are low and HCV services are underutilized despite a huge drop in the pricing of the treatment in the last year.

**Fig. 1:**
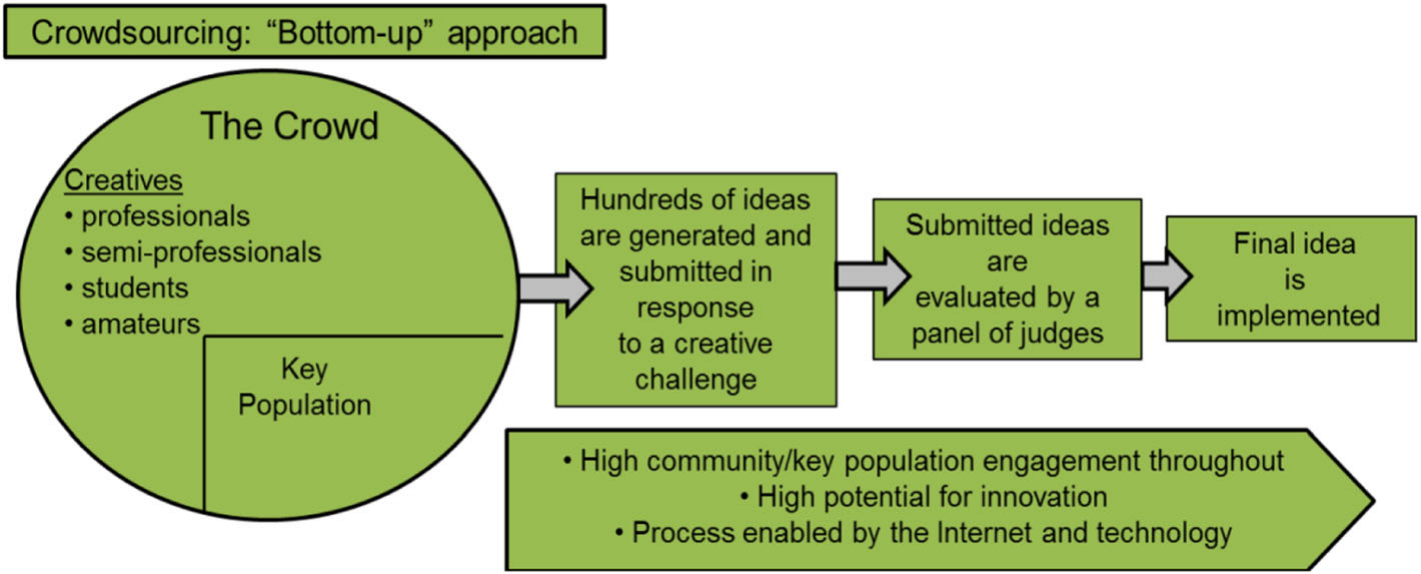
Crowdsourcing: the “bottom-up” approach to solicit ideas on HCV testing and linkage to care

Crowdsourcing is a form of community engagement which invites the public for brainstorming of ideas and then promotes the solutions with the public audience [10]. It has the potential to expand the role of primary care in promoting viral hepatitis testing and care continuum in China, which will improve healthcare access for the millions of individuals infected with HCV (see Fig. 1). Further, research has shown great beneficial effects by employing crowdsourcing technique in healthcare setting- materials developed through crowdsourcing increased Human Immunodeficiency Virus (HIV) testing uptake and condom use [11, 12]. In addition, social media campaigns that involve active community participation have been associated with higher rates of HIV and sexually transmitted infection (STI) testing compared to passive testing campaigns [13, 14]. Data from 100 Chinese individuals showed that a crowdsourced intervention of images and videos substantially increased HBV testing compared to the standard of care (37.5% HBV testing in the intervention arm, 14.3% testing in the standard of care arm, OR 3.60, 95% CI 1.10–11.74) [15].

### Objectives

The current study aims to compare the effects of a crowdsourcing intervention to the control on HCV test uptake among patients attending the primary care departments in a tertiary hospital in China. The hypothesis is that the crowdsourced intervention will be superior to the control in promoting HCV test uptake among patients attending the primary care departments in a tertiary hospital in China.

## Methods

### Study design

This study will include: a pilot study to test the feasibility of participant recruitment in a tertiary hospital in Shenzhen and the social media-based intervention delivered on smartphones; a randomized controlled trial (RCT) to investigate the effectiveness of a crowdsourced intervention compared to no intervention (standard-of-care) in increasing HCV test uptake and linkage-to-care i.e. patients found to be positive for HCV will be referred onto the Division of Gastroenterology of HKU-SZH for further management, if agreeable to the patients.

### Study setting

All phases of this study is conducted through the University of Hong Kong-Shenzhen Hospital (HKU-SZH) in Shenzhen, China. HKU-SZH was established in 2012 as an affiliated hospital of the University of Hong Kong. The hospital has comprehensive clinical services and reported 1.52 million outpatient visits and 65,856 inpatient discharges in 2019. With a “smart hospital” policy in which all appointment bookings, payments, and laboratory results can be processed by each patient’s personal smartphone, HKU-SZH is equipped to implement technology-based interventions.

The city of Shenzhen is located in southern China bordering Hong Kong. Internal migrants from across China are drawn to Shenzhen by the unique economic opportunities of this fast-growing city [16]. A meta-analysis found increased rates of infectious disease, in particular HCV, in Chinese rural-to-urban migrants [17], making Shenzhen a particularly crucial region for increasing viral hepatitis awareness and improving access to care.

We will work primarily with two outpatient departments at HKU-SZH: the Department of Family Medicine and Primary Care (FMPC) and the Division of Gastroenterology under the Department of Medicine. FMPC currently draws 100,000 patient visits per year from the municipality of Shenzhen, Guangdong as well as nearby provinces. The Division of Gastroenterology currently draws about 60,000 outpatient visits and 2500 inpatient discharges per year, including patients with chronic liver disease and viral hepatitis.

### Recruitment

Recruitment will continue until 1006 eligible subjects are enrolled. The primary recruitment sites will be FMPC clinic rooms, as well as the endoscopy suite of HKU-SZH, which performs 22,000 endoscopies per year. Patients will be recruited by physicians during their clinic or endoscopy suite visit. HCV screening at endoscopy suites in the United States has been shown to increase HCV testing uptake by threefold [18]. In China, there is evidence that the reported prevalence of HCV increases with age, and individuals above the age of 30 years have seen an increase in reported incidence from passive HCV detection [19].

### Eligibility criteria

Prior to enrollment, potential participants must first complete an informed consent form in Chinese language, which explains the purpose of the study as well as the rights and responsibilities of the subject participants. Participants are invited to participate if they are 30 years old or older, have Chinese nationality, and will reside in Shenzhen for the following month. Participants will be excluded if they self-report one of the followings: known chronic HCV infection, testing for serum anti-HCV in the past 12 months, current or planned pregnancy, currently undergoing complex medical treatment for other conditions. All eligible participants must provide a working unique mobile phone number and a WeChat account to be enrolled. Wechat is currently the most popular messaging social media app in China, with more than 1 billion active users and 45 billion daily text messages according to the statistics shown in [20].

### Sample size

The anti-HCV seroprevalence in China is estimated to be between 2.2 to 3.7%, giving an average of 3% prevalence of anti-HCV in the recruited population [21, 22]. The testing rate of conventional public health marketing methods (messages created by public health professionals) is approximately 35% [14]. If we conservatively assume crowdsourcing improves testing rates by 10%, together with 90% power and an alpha risk of 5%, a sample size of 1006 patients (*n* = 503 in each arm) will be required.

### Randomization and allocation

Once they have signed the consent form, participants will be randomly allocated on a 1:1 ratio to either the intervention or control group through a randomization procedure using permuted blocks. SAS software (Cary, North Carolina, USA) will be used to create the allocation sequence using PROC PLAN and RANUNI functions. Participants will be allocated sequentially in the order in which they are enrolled. This study will be not be blinded, since participants will know their randomization assignment based on whether they receive the intervention, and investigators will be aware of the randomization assignment.

### Intervention

The crowdsourcing intervention is composed of two parts: 1) promotional materials developed through a nation-wide open contest in China, 2) a series of mini crowdsourcing contests to solicit participant feedback to improve the promotional materials. The impact of crowdsourcing is expected through both receiving the crowdsourced messages and actively engaging with the message.

Upon enrollment, participants will be required to add the designated study account as a WeChat contact. The study account will be monitored and maintained by the researchers. The intervention materials—two videos and two images designed to increase awareness about HCV—will be delivered to participants over the course of 2 weeks as individual messages. The initial materials were developed through a nation-wide open contest in China, and was previously evaluated for hepatitis testing uptake in men-who-have-sex-with-men (MSM) in China [15].

Next, participants will be asked whether they have expertise in the following fields: healthcare, social media, or education. Participants who have expertise will be asked to reflect on the intervention based on their expertise, while participants without expertise will be encouraged to reflect based on personal experience. All participants will be invited to submit 50- to 200-character suggestion on how to improve the intervention materials. Submissions will be judged by a panel of health workers, researchers, and community advocates on the following: [1] potential to encourage HCV testing [2]; creativity [3]; potential to engage social media users; and [4] feasibility in clinical settings in Shenzhen. Suggestions for improvement will be shared anonymously among agreeable participants who are receptive to receive frequent updates from WeChat. Top scoring suggestions that meet the predesignated standard will receive cash prizes up to 500 Chinese yuan (72.5 USD). Feasible suggestions will be incorporated into the intervention materials and presented to the next round of participants.

Participants in the control arm will only receive the baseline and follow-up surveys via WeChat. They will not receive any promotional materials as there are currently no materials used in standard-of-care HCV testing promotion at the hospital. In the final week of the intervention period, all participants will receive a reminder to obtain HCV testing and a follow-up survey.

#### Linkage-to-care for HCV at HKU-SZH

During the four-week intervention period, participants tested positive for HCV antibody screening test will be referred to Department of Gastroenterology at the hospital. Blood tests will be collected for HCV confirmatory testing, and the participant will be informed of the test results within five working days. Those who have confirmed HCV infection via HCV RNA testing (Abbott Laboratories, lower limit of detection 15 IU/mL), respectively, will be referred to HKU-SZH’s specialty Liver Clinic in the Department of Gastroenterology for further diagnostic workup (liver biochemistry, ultrasonography, transient elastography, genotyping) and discussion of direct-acting antiviral treatment. Sovaldi-based treatment will be discussed, which will be replaced by Epclusa-based treatment when available. Patients will then be monitor for sustained virological response. After successful eradication, patients with cirrhosis or advanced fibrosis will continue follow-up at the HKU-SZH Liver Clinic, while those with non-advanced fibrosis will be referred to FMPC for follow-up. Participants that obtain anti-HCV testing or HCV RNA confirmatory testing at any facility in Shenzhen will be reimbursed for testing costs if they are able to provide medical records for confirmation.

#### Pilot study

Thirty participants will be recruited for the pilot to evaluate on the feasibility intervention delivery via WeChat and the participatory and iterative components of the intervention package. In addition, the pilot will inform the expected rate of recruitment, rate of participant retention from completion of the baseline survey to HCV testing uptake, and rate of linkage-to-care. At the end of the pilot period, participants will be asked to provide feedback on the trial design.

### Outcomes

The primary outcome is the number of anti-HCV screening tests completed by participants in either RCT arm within 4 weeks after enrollment. This dichotomous outcome will be determined by a single endpoint: uptake of HCV antibody screening test. Participants will only be counted as achieving the primary outcome if medical records at HKU-SZH or in another facility in Shenzhen confirm that they had tested for HCV during the intervention period. Medical records would be assessed based on either the administrative record or photo verification of official testing record.

Secondary outcomes are as follows: rate of participation in the crowdsourcing intervention; content of feedback submissions; number of participants that self-report HCV antibody testing but are not confirmed by medical records; number of participants that follow-up for HCV confirmatory testing; number of HCV diagnosed participants to continue with anti-HCV treatment, versus those who decline treatment for any reason; number of participants that will be completely cured of HCV infection, versus number that drop out prior to completing treatment; number of participants diagnosed with chronic liver disease (including cirrhosis, liver failure, or hepatocellular carcinoma); number of participants that will return to primary care for chronic liver disease follow-up or future screening of HCV and other conditions. Definitions for all study outcomes are provided in Table 1.

**Table 1:** Primary and secondary outcomes with definitions

**Primary Outcome**	**Definition**
Confirmed HCV testing uptake	Participants that complete HCV testing as confirmed by medical records at HKU-SZH or other public facility in Shenzhen within 4 weeks of enrollment
**Secondary Outcomes**	**Definitions**
Self-reported HCV testing uptake	Participants that complete HCV testing within 4 weeks of enrollment, self-reported in follow-up survey
Followed-up for HCV confirmatory testing	Participants that follow-up with a provider at HKU-SZH for HCV RNA diagnostic testing, as confirmed by medical records at HKU-SZH or other public facility in Shenzhen
Self-reported follow-up for HCV confirmatory testing	Participants that follow-up with a provider at HKU-SZH or other care facilities in China for HCV RNA confirmatory testing, self-reported in follow-up survey
Initiated anti-HCV treatment	Participants that have a confirmed HCV diagnosis and initiate anti-HCV treatment at HKU-SZH Department of Gastroenterology, confirmed by medical records at HKU-SZH or other public facility in Shenzhen
Completed anti-HCV treatment	Participants that complete the full anti-HCV regiment, as confirmed by medical records at HKU-SZH
Diagnosed with chronic liver disease	Participants that are diagnosed with chronic liver disease including cirrhosis, advanced cirrhosis, or hepatocellular carcinoma at any point during the study, as confirmed by medical records at HKU-SZH
Followed-up with Liver Clinic for advanced chronic liver disease	Participants that are diagnosed with advanced chronic liver disease (i.e. advanced cirrhosis) and receive follow-up care at the Liver Clinic at HKU-SZH, as confirmed by medical records at HKU-SZH
Cured of HCV infection	Participants that achieve sustained virological response (SVR), defined as absence of detectable hepatitis C virus 12 weeks after completion of treatment, confirmed by medical records at HKU-SZH
Returned to primary care for future screening	Participants that returned to FMPC for services including regular HBV/HCV screening, HBV screening, HIV screening, other STI screening, as confirmed by medical records at HKU-SZH

### Data collection and measures

Pre-intervention and post-intervention surveys will be delivered to the participants’ mobile devices and completed using Survey Star^@^ (Wechat, China), a web-based survey platform that meets industry standards for security and functionality. Both surveys were written in Chinese and verified for clarity and cultural appropriateness with 13 Chinese individuals in Guangdong province, including both genders and ages groups.

Recruited individuals will first be assessed for eligibility by the researcher. Eligible participants will then be asked to complete the baseline survey on Survey Star^@^. The baseline survey will include the following domains: sociodemographic characteristics, including migrant status; access to healthcare at HKU-SZH; trust in healthcare providers; knowledge of and attitudes towards HCV; and risk of contracting HCV. Migrant status will be defined based on the definition of internal migrant from the National Bureau of Statistics of China [23]. Trust in healthcare providers will be assessed using three items previously used to measure provider trust in young Chinese men [15]. Stigma towards HCV will be assessed using a combination of items adapted from the Toronto. Risk of contracting HCV was assessed using 8 items typically used by HKU-SZH providers for assessing HCV risk.

### Confidentiality

Baseline and follow-up survey data will be collected on Survey Star^@^. For follow-up on HCV testing results at HKU-SZH or in one of the connected public hospitals in Shenzhen, patient records in the electronic medical system will be traced by the research team using self-reported hospital registration ID and demographic information. All study data will be encrypted prior to transmission and will be stored in a secure server and can be access by login information known only to the research team. Ethics approval is obtained through the IRB of HKU-SZH (hkuszh201888).

### Incentives and compensation

Participants who participate in the study will receive an incentive totaling to 14.48 USD (100 RMB). During the testing period, the HCV screening test (serum anti-HCV antibody) will be reimbursed to participants in either study arm, after the participant completes the follow-up survey and the research team confirms the testing records. Reimbursements will cover up to 4 USD (28 RMB) of testing costs for HCV. The participant will receive reimbursement only if the demographic information on the test results matches the participant’s self-reported information, and the date of testing falls within the intervention period.

### Monitoring

A researcher will check each survey for completion and internal consistency. Each participant’s baseline and follow-up survey and test report will be checked for consistency of their verified cell phone number and demographic information. Test results must be confirmed by testing records at HKU-SZH or verified based on participant socio-demographics as reported in their baseline survey. A telephone number and WeChat account managed by the research team will be made available to participants for any questions or concerns that may come up during the study.

### Data analysis

#### Primary analysis

Participant baseline characteristics will be summarized using descriptive analyses. The primary analysis will evaluate whether the crowdsourced internet-based intervention package is superior to standard-of-care in increasing HCV testing uptake among the general public and target populations in China. The effect of the intervention on HCV testing uptake will be evaluated first using an intention to treat analysis that includes participants lost to follow-up. The proportion of participants with confirmed HCV testing in the intervention and control arms will be calculated separately using a 95% binomial proportion confidence interval (CI). The proportion of testing uptake between intervention and control arms will be compared using chi square test. Logistic regression adjusted with potential confounders will be applied to confirm the effect of intervention on the outcomes. Moreover, multiple logistic regression will be conducted to evaluate the effect of the following baseline factors on HCV testing uptake: previous health care service utilization behavior; trust in physicians and the health care system; knowledge of and access to healthcare services; membership in groups at high risk for HCV.

#### Missing data plan

Any participants lost to follow-up will be included. If an outcome is missing for < 15% of participants, analyses will use a complete-case approach. If an outcome is missing for ≥15% of participants, analyses will use multiple imputation.

#### Secondary analyses

Participants in the control and intervention arms will also be compared for differences in proportion of participants achieving secondary outcomes: number requiring referral for HCV confirmatory testing; number that receive confirmed diagnosis for HCV; number that attend follow-up visits at HKU-SZH for HCV treatment; number that achieve HCV cure.

## Discussion

Historically, HCV prevalence in China has been low, yet data suggests that that the HCV prevalence in China is rising across age groups [5, 24]. This may be attributed to that HCV currently does not have a preventive vaccine, and there is poor public awareness of the virus. As such, the HCV care continuum in China should be addressed urgently. However, according to a recent systematic review, few studies have evaluated interventions to increase HCV testing or treatment uptake [25]. Additionally, prior studies have targeted special populations such as people who use drugs (PWUD) and MSM, and have been limited to high risk group in high-income countries [25]. Few controlled trials have been conducted among the general population in low- or middle-income countries with high prevalence of viral hepatitis.

Our study is the first to utilize crowdsourcing for viral hepatitis testing and linkage-to-care in a general population. Our study be conducted in context of the new and evolving primary care system in China, a middle-income country that is endemic for HCV, but where public and professional knowledge about HCV is limited. The findings will contribute significantly to the existing literature on viral hepatitis testing in the primary care setting.

Crowdsourced images promoted through social media platforms have demonstrated efficacy for increasing HIV screening and condom use while simultaneously increasing community engagement and knowledge about the respective issue [11, 12]. Crowdsourcing also has the potential to address sensitive issues associated with marginalized populations such as MSM [13]. However, despite the high rate of social media and technology use China crowdsourcing may have limited reach to certain individuals. These overlooked individuals are likely to be part of the vulnerable population such as PWUD or people with mental health disorders, or older people with low technological literacy or social media use. Since the participation in our study requires the use of a smartphone and knowledge of social media, the study will exclude those who do not use these services. The recruitment of the targeted study population based on an establishing hospital setting would form a limitation as participants would have been engaged in medical care or specific treatment. Individuals who are distrustful of or disengaged from medical care are less likely to be recruited. Finally, our study does not directly address the lack of primary provider training in China’s HCV care continuum [9].

The findings from this study can still inform future decisions and policies to improve HCV care training for primary care providers in China and shape the future role of China’s primary care system in public health campaigns.

## Data Availability

Currently not available as the trial is still in progress. Future data from this trial can be obtained from the corresponding author on reasonable request.

## Abbreviations

HCV: Hepatitis C virus
HIV: Human immunodeficiency virus
STI: Sexually transmitted infections
HKU-SZH: University of Hong Kong-Shenzhen Hospital
FMPC: Department of family medicine and primary care
RCT: Randomized controlled trial
WHO: World health organization
SVR: Sustained virological response
PWUD: People who use drugs
MSM: Men who have sex with men

## References

[1] Global prevalence and genotype distribution of hepatitis C virus infection in 2015: a modelling study. Lancet Gastroenterol Hepatol. 2017;2(3):161–76. 10.1016/S2468-1253(16)30181-9 28404132

[2] Organization WHO. WHO guidelines on hepatitis B and C testing. Geneva: World Health Organization; 2017. Licence: CC BY-NC-SA 3.0 IGO.10. Organization WHO. Crowdsourcing in health and health research: a practical guide. Geneva: World Health Organization; 2018. TDR/STRA/18.4. Licence: CC BY-NC-SA 3.0 IGO.

[3] Thomas DL (2013). Global control of hepatitis C: where challenge meets opportunity. Nat Med.

[4] Liu CR, Li X, Chan PL, Zhuang H, Jia JD, Wang X (2019). Prevalence of hepatitis C virus infection among key populations in China: a systematic review. Int J Infect Dis.

[5] Bennett H, Waser N, Johnston K, Kao JH, Lim YS, Duan ZP (2015). A review of the burden of hepatitis C virus infection in China, Japan, South Kore and Taiwan. Hepatol Int.

[6] Carrat F, Fontaine H, Dorival C, Simony M, Diallo A, Hezode C (2019). Clinical outcomes in patients with chronic hepatitis C after direct-acting antiviral treatment: a prospective cohort study. Lancet (London, England).

[7] Li M, Zhuang H, Wei L (2019). How would China achieve WHO's target of eliminating HCV by 2030?. Expert Rev Anti-Infect Ther.

[8] Liu L, Xu H, Hu Y, Shang J, Jiang J, Yu L (2019). Hepatitis C screening in hospitals: find the missing patients. Virol J.

[9] Wong WCW, Lo YR, Jiang S, Peng M, Zhu S, Kidd MR, et al. Improving the hepatitis cascade: assessing hepatitis testing and its management in primary health care in China. Fam Pract. 2018. 10.1093/fampra/cmy032 29741661

[10] Organization WH, UNICEF. Crowdsourcing in health and health research: a practical guide: World Health Organization; 2018.

[11] Liu C, Mao J, Wong T, Tang W, Tso LS, Tang S (2016). Comparing the effectiveness of a crowdsourced video and a social marketing video in promoting condom use among Chinese men who have sex with men: a study protocol. BMJ Open.

[12] Tang W, Wei C, Cao B, Wu D, Li KT, Lu H (2018). Crowdsourcing to expand HIV testing among men who have sex with men in China: a closed cohort stepped wedge cluster randomized controlled trial. PLoS Med.

[13] Cao B, Liu C, Durvasula M, Tang W, Pan S, Saffer AJ (2017). Social media engagement and HIV testing among men who have sex with men in China: a Nationwide cross-sectional survey. J Med Internet Res.

[14] Tang W, Han L, Best J, Zhang Y, Mollan K, Kim J (2016). Crowdsourcing HIV test promotion videos: a noninferiority randomized controlled trial in China. Clin Infect Dis.

[15] Fitzpatrick T, Zhou K, Cheng Y, Chan P-L, Cui F, Tang W (2018). A crowdsourced intervention to promote hepatitis B and C testing among men who have sex with men in China: study protocol for a nationwide online randomized controlled trial. BMC Infect Dis.

[16] 陈东平. 深圳市人口结构分析报告. 深圳社会建设与发展报告. 深圳蓝皮书2016. p. 284–6.

[17] Zou X, Chow EP, Zhao P, Xu Y, Ling L, Zhang L (2014). Rural-to-urban migrants are at high risk of sexually transmitted and viral hepatitis infections in China: a systematic review and meta-analysis. BMC Infect Dis.

[18] Campbell B, Liu B, Bhuket T, Wong RJ (2018). Pilot study of screening patients for hepatitis C virus infection during outpatient endoscopy. Clin Gastroenterol Hepatol.

[19] Qin Q, Smith MK, Wang L, Su Y, Wang L, Guo W (2015). Hepatitis C virus infection in China: an emerging public health issue. J Viral Hepat.

[20] Wechat. 2018 Wechat's annual data report. https://support.weixin.qq.com/cgi-bin/mmsupport-bin/getopendays. Accessed 15 June 2020.

[21] Lavanchy D (2011). Evolving epidemiology of hepatitis C virus. Clin Microbiol Infect.

[22] Wei L, Lok AS (2014). Impact of new hepatitis C treatments in different regions of the world. Gastroenterology.

[23] Statistical Communiqué of the People's Republic of China on the 2016 National Economic and social development. National Bureau of Statistics of China; 2017.

[24] Liu Z, Yang Q, Shi O, Ye W, Chen X, Zhang T. The epidemiology of hepatitis B and hepatitis C infections in China from 2004 to 2014: An observational population-based study. J Viral Hepat. 2018;25(12):1543–54. 10.1111/jvh.12938 29851287

[25] Zhou K, Fitzpatrick T, Walsh N, Kim JY, Chou R, Lackey M (2016). Interventions to optimise the care continuum for chronic viral hepatitis: a systematic review and meta-analyses. Lancet Infect Dis.

